# Voxel-based mapping of grey matter volume and glucose metabolism profiles in amyotrophic lateral sclerosis

**DOI:** 10.1186/s13550-017-0267-2

**Published:** 2017-03-06

**Authors:** M-S. Buhour, F. Doidy, A. Mondou, A. Pélerin, L. Carluer, F. Eustache, F. Viader, B. Desgranges

**Affiliations:** Normandie Univ, UNICAEN, EPHE, INSERM, U1077, CHU de Caen, Neuropsychologie et Imagerie de la Mémoire Humaine, 14000 Caen, France

**Keywords:** Amyotrophic lateral sclerosis, Magnetic resonance imaging, Positron emission tomography, Voxel-based morphometry, Hypermetabolism

## Abstract

**Background:**

Amyotrophic lateral sclerosis (ALS) is a rapidly progressive disease of the nervous system involving both upper and lower motor neurons. The patterns of structural and metabolic brain alterations are still unclear. Several studies using anatomical MRI yielded a number of discrepancies in their results, and a few PET studies investigated the effect of ALS on cerebral glucose metabolism. The aim of this study was threefold: to highlight the patterns of grey matter (GM) atrophy, hypometabolism and hypermetabolism in patients with ALS, then to understand the neurobehavioral significance of hypermetabolism and, finally, to investigate the regional differences between the morphologic and functional changes in ALS patients, using a specially designed voxel-based method.

Thirty-seven patients with ALS and 37 age- and sex-matched healthy individuals underwent both structural MRI and ^18^[F]-fluorodeoxyglucose (FDG) PET examinations. PET data were corrected for partial volume effects. Structural and metabolic abnormalities were examined in ALS patients compared with control subjects using two-sample *t* tests in statistical parametric mapping (SPM). Then, we extracted the metabolic values of clusters presenting hypermetabolism to correlate with selected cognitive scores. Finally, GM atrophy and hypometabolism patterns were directly compared with a one-paired *t* test in SPM.

**Results:**

We found GM atrophy as well as hypometabolism in motor and extra motor regions and hypermetabolism in medial temporal lobe and cerebellum. We observed *negative* correlations between the metabolism of the right and left parahippocampal gyri and episodic memory and between the metabolism of right temporal pole and cognitive theory of mind. GM atrophy predominated in the temporal pole, left hippocampus and right thalamus, while hypometabolism predominated in a single cluster in the left frontal superior medial cortex.

**Conclusions:**

Our findings provide direct evidence of regional variations in the hierarchy and relationships between GM atrophy and hypometabolism in ALS. Moreover, the ^18^FDG-PET investigation suggests that cerebral hypermetabolism is deleterious to cognitive function in ALS.

## Background

Amyotrophic lateral sclerosis (ALS) is a neurodegenerative disease that primarily affects motor function but also concerns extramotor systems. The degeneration of the motor system typically involves both upper motor neurons, located in the primary motor cortex, and lower motor neurons from the brainstem nuclei and anterior horns of the spinal cord. The disease has a uniformly fatal outcome as a result of muscle weakness, with median survival of 2-4 years [1]. The underlying pathophysiology is poorly understood, and effective treatments are still needed for this neurodegenerative disease.

There is increasing awareness that ALS is a clinically heterogeneous disease [2]. There is also a general recognition that ALS patients commonly present deficits not only in executive functions but also memory [3, 4] and social cognition with impairment of both cognitive and affective theory of mind [5–7].

Magnetic resonance imaging (MRI) of the brain and spinal cord is routinely used in the diagnostic work-up of ALS, to rule out various pathological conditions that may masquerade as motor neuron disease, but rarely gives specific clues to the positive diagnosis. Automated techniques for analysing MRI images have been developed notably with statistical parametric mapping (SPM) software to carry out voxel-by-voxel analysis of the whole brain (voxel-based morphometry (VBM)). Most of the studies, have reported extensive grey matter (GM) atrophy, not confined to motor areas [8].

Positron emission tomography (PET) combined with ^18^F-fluorodeoxyglucose (FDG), a specific radiotracer for glucose metabolism, indicates glucose uptake by astrocytes and neurons [9] and reveals local brain activity. Early studies found reduced regional cerebral glucose utilization in patients with ALS [10], mostly in the frontal cortex but also in other cortical territories such as the superior occipital cortex [11]. More recently, other authors, adopting a voxel-by-voxel approach [12–16], have observed severe hypometabolism in the premotor cortex, postcentral gyrus, prefrontal cortex, lingual gyrus, fusiform gyrus and thalamus. They also observed an increased cerebral glucose metabolism or hypermetabolism in the medial temporal lobe, cerebellum, occipital cortex and brainstem. This hypermetabolism has been proposed as a possible biomarker for ALS by Pagani and colleagues [13]. It is not yet known, however, whether it reflects a compensatory mechanism in the brain or deleterious metabolic activity. Nevertheless, it is important to note that the limited PET camera resolution can give rise to partial volume effects (PVEs) that result in blurring and, therefore, an underestimation of regional activity, particularly in small structures or those with volume loss. Since previous FDG-PET studies did not correct for PVEs, the metabolic abnormalities observed in patients with ALS have to be interpreted cautiously.

The abovementioned studies investigated different groups of non-demented patients with ALS using either brain MRI or FDG-PET (without correction for PVEs). Only one combined these two techniques in a single group of 18 patients with ALS who met the Neary criteria for frontotemporal dementia (FTD) [17]. The authors assessed brain GM structural changes using VBM and metabolic changes using FDG-PET. They concluded that the metabolic changes corresponded to the structural changes, with a few exceptions.

To our knowledge, no combined assessment of GM volume and regional cerebral metabolism has yet been carried out in non-demented patients with ALS. The goals of the present study were threefold: (1) determine both GM volume and glucose metabolism changes in a sample of patients with ALS by adopting a voxel-based approach; (2) further study the clinical significance of hypermetabolism by assessing its relationship with selected cognitive scores; and (3) carry out a direct voxelwise comparison of the degrees of local GM atrophy and hypometabolism throughout the brain by using a specific processing technique developed in our laboratory that has already been applied in early Alzheimer’s disease [18], alcoholism [19] and the behavioural variant of FTD [20].

## Methods

### Participants

#### Participants with ALS

Participants were 37 patients with ALS (21 men and 16 women; mean age = 61.18 years, standard deviation = 11.11; mean level of education = 10.03 years, standard deviation = 2.96) with either bulbar (*n* = 10) or spinal (*n* = 27) onset. Patients with ALS were recruited between November 2009 and June 2014 via the ALS clinic of the neurology department of Caen University Hospital (France). All participants were native French speakers and had a minimum level of education equivalent to a now obsolete school-leaving certificate that was generally taken in 14 years. All the patients met the modified El Escorial criteria for probable or definite ALS [21]. Exclusion criteria were the additional presence of severe and chronic illness, alcohol or drug abuse, traumatic brain injury or an extremely severe communication problem that could seriously compromise the administration of cognitive tests. None of the patients fulfilled the criteria for a diagnosis of FTD according to the core and supportive diagnostic features of FTD detailed in Lund and Manchester’s consensus statement [22]. None of the patients with ALS met the criteria for a possible and/or probable behavioural variant of FTD [23]. Behavioural disorders were explored in 32 patients via the short form of the Neuropsychiatric Inventory [24]. None had behavioural disorders of the frontal type sufficiently severe to meet the criteria for ALS with behavioural impairment. All the patients underwent a neurological assessment that included the ALS Functional Rating Scale Revised (ALS-FRS-R) [25], Norris scale [26] and Medical Research Council (MRC, 1976) Muscle Strength Scale. They were able to speak or to write intelligibly. All the patients gave their written informed consent, and the independent regional ethics committee approved the study. Some of these patients had already been included in a previous study [5]. The ALS patients underwent a neuropsychological assessment covering a range of cognitive functions (for details, see [5]).

#### Control groups

##### Neuroimaging

To compare the neuroimaging data, we included a group of 37 healthy controls (21 men and 16 women; mean age = 61.11 years, standard deviation = 11.11; mean level of education = 11.45 years, standard deviation = 2.97). Healthy controls were recruited from the community and performed in the normal range on a neuropsychological examination assessing multiple domains of cognition including episodic and semantic memory, executive and visuospatial functions, language and praxis. Within a few days from recruitment, they also underwent a structural MRI and a PET using [^18^F] fluoro-2-deoxy-D-glucose (^18^FDG). These controls were closely matched for sex, age and level of education with the group of patients with ALS.

##### Cognition

As most of our neuropsychological tests were original, with no available normative values, to compare the cognitive scores, we included another group of 37 healthy subjects (21 men and 16 women; mean age = 61.81 years, standard deviation = 8.84; mean level of education = 11.19 years, standard deviation = 2.60). The Mattis cut-off score was set at 130 to avoid the possible inclusion of controls with mild cognitive impairment (for details, see Table 1).

**Table 1:** Demographic, medical and cognitive data of controls and patients with ALS

	Controls (cognition)	Controls (imaging)	ALS patients	*p*
Main features of the participants				
Age (years)	61.81	59	61.83	0.83 (cognition)/0.91 (imaging)
Sex (F/M)	16/21	16/21	16/21	
Years of education	11.11	11.43	9.92	0.070 (cognition)/0.099 (imaging)
Mattis (total)	139.95	141	136.47	0.005 (cognition)/<0.001 (imaging)
ALSFRS-*R* score	/	/	37.31	
Cognitive profile				
TMT (B–A)	15.94		62.97	<0.001
Letter verbal fluency score	22.24		15.03	<0.001
Letter verbal fluency index	4.71		7.68	0.017
Episodic memory	12.14		7.08	<0.001
Cognitive theory of mind	13.22		11.36	0.009
Affective theory of mind	12.44		11.03	0.062

### Neuropsychological assessment

Patients and controls underwent a neuropsychological assessment that evaluated a set of cognitive functions that were already described and published in a previous study (for details, see [5]). We explored the affective and cognitive theory of mind, the episodic memory and the executive functions abilities of ALS patients. Affective theory of mind was assessed with the Reading the Mind in the Eyes test derived from the procedure used by Baron-Cohen et al. [27]. The participants had to make inferences about the affective and motivational states of others on the basis of a picture of their eyes (for more details, see [28]). Cognitive theory of mind abilities were assessed with the TOM-15 task, a false-belief task which comprises 15 short comic strips (for details, see [5]). We then explored episodic memory, with a task in which participants had to intentionally encode a list of eighteen unrelated words. Every three words, they were subjected to an immediate free recall task. Following the study phase, there was a 20-second interval during which participants were engaged in an unrelated distractor task. Participants then had to recall as many words as possible, with no time limit. After a 20-min interval, they were finally subjected to a delayed free recall task, again, with no time limit. Finally, executive functions were assessed with the Trail Making Test (TMT), and a letter verbal fluency task (raw score and index, taking into account the effect of disease-induced motor impairment (for details, see [5]).

### Neuroimaging data acquisition

All participants were scanned using the same MRI and PET cameras at the Cyceron centre (Caen, France): a Philips Achieva 3.0 T scanner and a discovery RX VCT 64 PET-CT device (General Electric Healthcare), respectively.

#### MRI data

For each participant, a high-resolution T1-weighted anatomical image was acquired on a Philips Achieva 3 T scanner using a three-dimensional fast field echo sequence (sagittal; repetition time = 20 ms, echo time = 4.6 ms, flip angle = 10°, 180 slices, slice thickness = 1 mm, field of view = 256 × 256 mm^2^, matrix = 256 × 256).

#### PET data

Each participant underwent a PET examination the day after MRI examination. FDG scans were acquired on a Discovery RX VCT 64 PET-CT device (GE Healthcare) with a resolution of 3.76 × 3.76 × 4.9 mm^3^ FWHM (axial field of view = 157 mm). Forty-seven planes were obtained with septa out (3D acquisition), with a voxel size of 2.7 × 2.7 × 3.27 mm^3^. A CT transmission scan was performed for attenuation correction before PET acquisition. Participants were fasted for at least 6 h before scanning. After a 30-min resting period in a quiet and dark environment, ~180 MBq of FDG were intravenously injected as a bolus. A 10-min PET acquisition scan began 50 min after injection. During data acquisition, head motion was continuously monitored with laser beams projected onto ink marks drawn on the forehead, which also served to make any necessary corrections.

### Neuroimaging data handling and transformation

#### Preprocessing

Using the VBM5.1 toolbox (http://dbm.neuro.uni-jena.de/vbm/vbm5-for-spm5/), implemented in SPM5 software (Wellcome Trust Centre for Neuroimaging, London, UK), the raw MRI data were spatially normalized to Montreal Neurological Institute (MNI) space (voxel size = 1 mm^3^, matrix = 156 × 189 × 157) and segmented into the GM, white matter (WM) and cerebrospinal fluid (CSF). The normalized GM images were modulated by the Jacobian determinants to correct for nonlinear warping only, to obtain maps of local GM volumes corrected for brain size.

FDG-PET data were first corrected for CSF and WM PVEs, using the voxel-by-voxel modified Müller-Gartner method [29, 30], described in detail elsewhere [31], and already used in our laboratory [18–20]. Using SPM5, the PVE-corrected PET data were then coregistered (rigid-body coregistration) to their respective native MRIs and normalized to MNI space by reapplying the normalization parameters estimated from the VBM protocol described above. The resulting images underwent quantitative scaling using mean GM as a reference.

#### Smoothing

For the between-group comparison, and in order to blur individual variations in gyral anatomy and increase the signal-to-noise ratio, the registered MRI data were smoothed using an 8 × 8× 8 (*x*, *y*, *z*)-mm Gaussian kernel. PET datasets were also smoothed, using a 12 × 12 × 12-mm Gaussian kernel.

#### Differential smoothing

For the direct comparison between GM atrophy and glucose hypometabolism, since the MRI and PET data had different spatial resolutions, differential smoothing was applied to equalize the effective smoothness [32–34]. A Gaussian kernel of 8 × 8 × 8 (*x*, *y*, *z*) mm was used for the MRI data and 7.1 × 7.1 × 6.4 for the PET data. Finally, images were masked to exclude non-GM voxels from the analysis.

#### *W* score maps

To obtain measurements of atrophy and hypometabolism expressed in the same units so that we could undertake direct comparisons of the different modalities, we computed *W* score maps for each patient and each imaging modality, using the healthy control group as a reference. *W* scores are analogous to *z* scores but are adjusted for specific covariates [35]—age in the present case. The smoothed MRI and PET data were used to create *W* score maps [(raw value for each patient) − (value expected in the control group for the patient’s age)/(*SD* for each patient and each modality); see [18] for more details].

As the two datasets had different original spatial resolutions, the MRI data were coregistered and resliced into PET space to obtain images with the same dimensions.

#### Anatomical localization

GM anatomical localization was carried out using AAL automated labelling software implemented in SPM5 [36].

### Statistical analyses

#### Between-group comparison of GM volume and metabolism

To determine the pattern of GM atrophy in the ALS group as well as glucose hypometabolism and hypermetabolism, compared to the control group, we used a two-sample *t* test in SPM5. This yielded three maps of statistically significant GM atrophy and metabolic abnormalities in patients relative to controls. To address the issue of multiple comparisons, for each analysis, the significant cluster size was determined using a Monte Carlo simulation program (AlphaSim) as previously done in our laboratory [37, 38], with a cumulative proportion criterion of less than 0.05 [39]. For the GM volume analysis, this equated to a cluster volume (=k) of 475 voxels and for the metabolism analyses a *k* = 113 voxels.

#### Correlations between hypermetabolism and selected cognitive scores

Statistical analyses were performed using Statistica 10.0 (StatSoft, Tulsa, OK, USA). The threshold of significance was set at *p* = .05 (one-tailed). We assessed correlations between the metabolism of significant clusters identified in the previous analysis showing hypermetabolism and selected cognitive tests. To this end, we extracted the mean metabolic values of the regions presenting hypermetabolism using the “binary ROIs analysis” in SPM5 and then entered them into Statistica to assess correlations with cognitive scores of the patients with ALS.

#### Within-group comparison between morphological and metabolic alterations

Individual GM MRI *W* score maps and PET *W* scores maps were entered in a one-paired *t* test analysis with one group (patients with ALS) and two images per participant (i.e. MRI and PET *W* score maps), using SPM5. We put years of education and Mattis (total score) as confounding covariates in this analysis. Both contrasts were assessed (*W*-PET > W-MRI and *W*-MRI > *W*-PET) to generate statistical maps reflecting predominant structural abnormalities or glucose hypometabolism. To address the issue of multiple comparisons, significant cluster size was determined using a Monte Carlo simulation program (AlphaSim), with a cumulative proportion criterion of less than 0.05 [39]. This equated to a cluster volume of 31 voxels.

## Results

### Behavioural results

Regarding executive functions, compared with the control group, the patients exhibited significant impairment of executive functions on the Trail Making Test B–A (*p* < 0.001), letter verbal fluency score (*p* < 0.001) and letter verbal fluency index (*p* = 0.017). The same was true for both episodic memory, measured with a classic word learning test (*p* < 0.001), and cognitive theory of mind, assessed with a false-belief task (*p* = 0.009), with a trend for affective theory of mind, assessed with the Face/Eyes test (*p* = 0.062) (for details, see Table 1). When comparing ALS patients with bulbar onset to those with spinal onset, we did not find any difference in their cognitive status (data not shown).

### Patterns of grey matter atrophy and hypometabolism in ALS

**Fig. 1:**
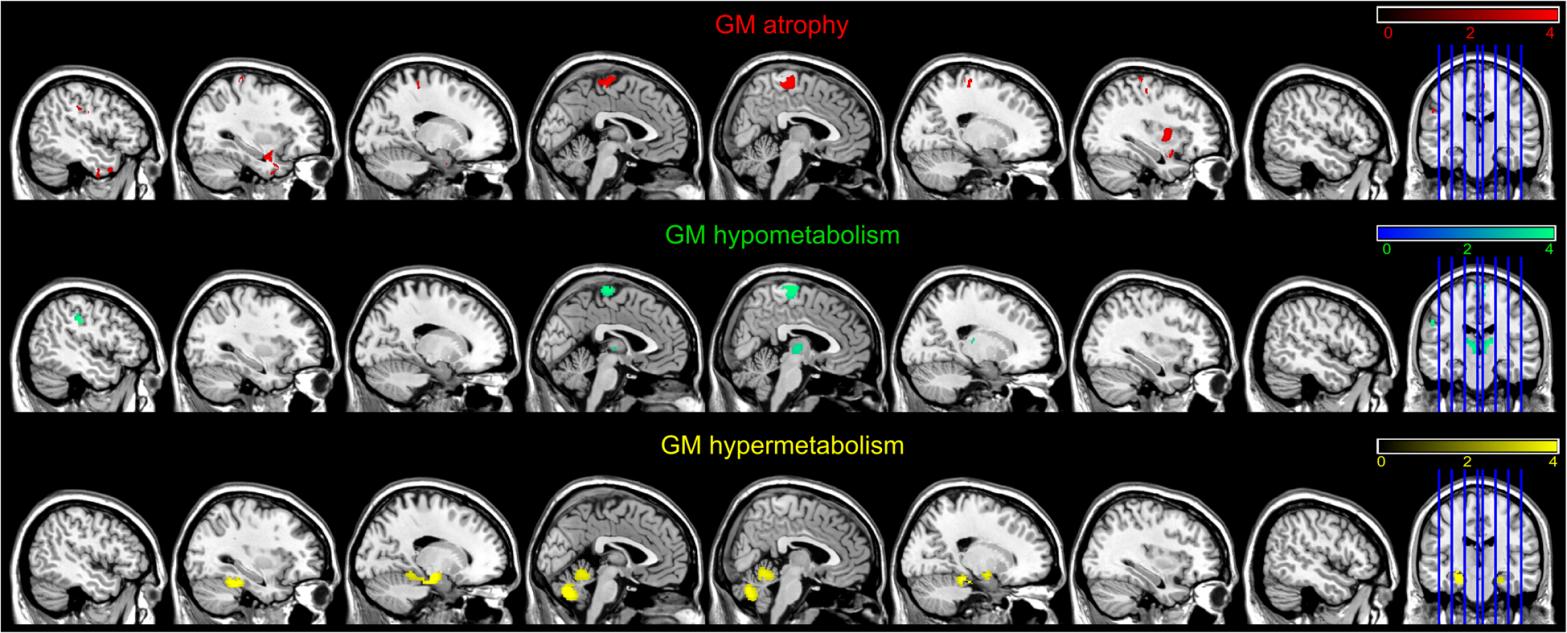
GM atrophy (in *red*), hypometabolism in (*green*) and hypermetabolism (in *yellow*) in patients withamyotrophic lateral sclerosis compared with healthy controls. Between-group comparisons were conductedwith the SPM5 two-sample t test routine. We used a *p* value cutoff of *p* < 0.001 uncorrected for multiple comparisons

The comparison of GM volume between patients and controls revealed significant GM atrophy in the left postcentral gyrus, left inferior temporal gyrus, left paracentral lobule and left supramarginal gyrus (see Fig. 1). We also found GM atrophy within the right precentral gyrus and right putamen.

Hypometabolism mainly concerned the right paracentral lobule, the left inferior parietal gyrus, the thalamus bilaterally as well as the left superior medial frontal gyrus.

Patients also showed hypermetabolism mainly in the cerebellar vermis (IV, V and VII), cerebellar lobules IV and V, bilaterally within the medial temporal cortex (both the hippocampus and parahippocampal gyri) and, to a lesser extent, the fusiform gyrus. All the results are shown in Fig. 1 and listed in Table 2.

**Table 2:** Labellization. MNI coordinates. Cluster size in number of voxels (K) and *T* value of the significant peaks for the SPM analyze of GM atrophy, hypometabolism, hypermetabolism and GM atrophy > hypometabolism and hypometabolism > GM atrophy

Labels	K	MNI coordinates			*p* corrected at the cluster level	*p* FWE corrected at voxel level	*T* (voxel level)
		*x*	*y*	*z*			
GM atrophy							
L postcentral	569	−53	−12	29	0.518	0.036	5.18
L inferior temporal gyrus	3146	−27	9	−33	0.002	0.102	4.86
L paracentral lobule	1331	−4	−27	64	0.084	0.133	4.77
L supramarginal gyrus	484	−52	−22	31	0.621	0.159	4.71
R precentral gyrus	1115	16	−26	67	0.14	0.541	4.23
R putamen	771	35	0	4	0.323	0.785	3.99
Hypometabolism							
R paracentral lobule	218	4	−22	70	0.217	0.032	5.21
L inferior parietal gyrus	142	−50	−24	34	0.398	0.099	4.26
R thalamus	244	8	−16	4	0.176	0.31	3.92
L superior medial frontal gyrus	142	−8	40	36	0.398	0.314	3.91
L thalamus	144	−8	−18	6	0.392	0.564	3.87
Hypermetabolism							
L hippocampus	868	−22	−18	−18	0.003	0.008	5.33
L parahippocampus							
L fusiform							
Vermis VII	941	−2	−72	−32	0.002	0.044	4.82
Vermis IV–V							
R fusiform	557	22	−32	−20	0.019	0.163	4.39
R cerebellum IV–V							
R parahippocampal gyrus							
R hippocampus							
Atrophy > hypometabolism							
L parahippocampus	147	−26	4	−30	0	0.709	4.85
L middle temporal pole							
L calcarine	36	−4	−96	8	0.109	0.784	4.76
R middle temporal pole	58	46	12	−26	0.013	0.936	4.52
L inferior temporal gyrus	87	−50	−6	−32	0.001	0.997	4.2
L hippocampus	56	−30	−12	−22	0.016	1	4.05
Hypometabolism > atrophy							
L superior medial frontal gyrus	48	−6	34	40	0.033	1	4.06

In our quest to better understand this metabolic dysfunction and investigate its neurobehavioural significance, we assessed the relationships between hypermetabolism and selected cognitive functions. Given that the medial temporal lobe is involved in episodic memory, the cerebellum is part of a motor network and fusiform gyrus is involved in social cognition [40, 41], we looked for positive or negative correlations between the metabolism of each of these regions and the relevant (and available) cognitive performances. All the significant correlations we found were negative. Thus, we observed significant negative correlations between episodic memory (immediate recall) and the metabolic value of the right hippocampus (*r* = −0.36; *p* = 0.04), left hippocampus (*r* = −0.36; *p* = 0.04) and left parahippocampal gyrus (*r* = −0.46; *p* = 0.007). Concerning the delayed recall, we found significant negative correlations with the metabolic values of the left parahippocampal gyrus (*r* = −0.45; *p* = 0.01). We also found negative correlations between metabolic activity within the left fusiform gyrus and TOM-15 (cognitive theory of mind) (*r* = −0.33; *p* = 0.04) (Table 3).

**Table 3:** Correlations between the metabolism of each region presenting hypermetabolism and the relevant cognitive performances in ALS patients.

	Episodic memory (immediate recall)	Episodic memory (delayed recall)	TOM-15
L hippocampus	*r* = −0.36; *p* = 0.045	*r* = −0.29; *p* = 0.105	
R hippocampus	*r* = −0.36; *p* = 0.044	*r* = −0.32; *p* = 0.077	
L parahippocampus	*r* = −0.46; *p* = 0.007	*r* = −0.45; *p* = 0.010	
R parahippocampus	*r* = −0.19; *p* = 0.289	*r* = −0.11; *p* = 0.564	
L fusiform			*r* = −0.3327; *p* = 0.044
R fusiform			*r* = −0.1324; *p* = 0.435

*R* right, *L* left

### Direct comparison between GM atrophy and hypometabolism in ALS patients

GM atrophy was greater than hypometabolism in the temporal lobes (left and right middle temporal poles, left hippocampus and left parahippocampus as well as left inferior temporal gyrus). This was also true in the left calcarine (Table 2).

The reverse contrast (hypometabolism > GM atrophy) only concerned the left superior medial frontal cortex.

## Discussion

To our knowledge, this was the first neuroimaging study to closely examine the relationship between cerebral GM volume and glucose metabolism via a direct comparison of both parameters, as well as the relationship between hypermetabolism and cognitive performance in patients with ALS. We first compared GM volume and cerebral glucose metabolism in patients versus controls. We found (1) marked GM atrophy not only in the right premotor cortex but also in extramotor regions, (2) glucose hypometabolism mainly in both the thalamus and parietal lobe and (3) glucose hypermetabolism located bilaterally in the cerebellar vermis, medial temporal lobe and fusiform gyrus. We then extracted the metabolic values of the main hypermetabolic clusters and correlated them with cognitive scores that were expected to depend on these brain regions. We found only negative correlations: first, between the hippocampus and left parahippocampus and episodic memory and second, between the left fusiform gyrus and cognitive theory of mind. We also conducted a voxelwise comparison of the degrees of local atrophy and hypometabolism, using a method specially designed for this purpose, and we observed that GM atrophy predominated in the bilateral middle temporal pole and left hippocampus, while hypometabolism predominated in a single cluster located in the left superior medial frontal cortex.

### Between-group comparisons

The profile of GM atrophy in our patients was in agreement with previous studies that had reported motor and extramotor GM atrophy in a parietotemporal network [42–49], as well as the putamen [44, 50–52].

The pathological involvement of the putamen in ALS may seem surprising, as patients do not typically exhibit extrapyramidal signs. However, ubiquitin immunoreactive intracytoplasmic inclusions and neuronal loss have been found in the striatonigral system in half of patients with classic motor neuron disease [53]. Interestingly, in the study of Kim et al., the putamen was among the most atrophied brain regions in cognitively impaired ALS patients [52]. Furthermore, the putamen has a major role in verbal fluency, working memory and speech production [54], cognitive domains frequently impaired in ALS [55]. Finally, putaminal regions receive afferences from the motor and premotor cortices [51], all of which being heavily affected in this study.

Concerning the hypometabolism, our results are in line with most previous studies [12–14, 16]. Concerning the hypometabolism in the paracentral lobule, this result has rarely been found with ^18^FDG-PET. Kew et al., using C^15^O_2_ in 12 patients with ALS, observed a significant reduction of cerebral blood flow at rest within this structure [56]. Concerning the decreased uptake of ^18^FDG within the left inferior parietal gyrus found in our sample of patients, PET studies using [^11^C]-flumazenil, or C^15^O_2_ [56–58], also found reduced regional cerebral blood flow within the parietal lobe. We also found significantly lower frontal metabolism (superior medial) in our patient group that is in line with the fact that they present frontal dysfunction. A study went further showing that prefrontal hypometabolism was associated with reduced clinical functioning in ALS patients [14]. Decreased glucose uptake in the thalamus has also been reported by Cistaro et al. and is thought to be a metabolic signature of *C9orf72*-related ALS [59]. Up to 10% of patients with ALS have a mutated gene; the most common of which is *C9orf72*. In patients with this mutation, the thalamic hypometabolism is slightly more marked than it is in sporadic ALS cases [14].

Our results confirm the brain hypermetabolism in ALS already demonstrated in previous studies [12–14, 16]. Concerning the brain regions that were found to exhibit increased glucose metabolism, results were mainly in agreement with previous studies that also observed increased glucose metabolism in the medial temporal lobe and cerebellum [13, 14].

### Negative correlations with episodic memory and cognitive theory of mind

We observed significant negative correlations between episodic memory (immediate recall) and the metabolic value of the right hippocampus, the left hippocampus and the left parahippocampal gyrus. We also found negative correlations between metabolic activity within the left fusiform gyrus and cognitive theory of mind. The negative correlation between metabolism and cognitive scores means that hypermetabolism is associated with a functional deficiency of the involved brain area. Indeed, given that our patients with ALS scored more poorly than controls for episodic memory as well as for cognitive theory of mind, this result indicates that the abnormal pattern of glucose metabolism within these regions was not associated with preserved performances. This result was not expected because generally, when studies found hypermetabolism or hyperactivation, it is usually considered as the reflection of compensatory mechanisms [60, 61]. However, within the framework of Huntington’s disease, a previous work has shown a link between hyperactivation of the precuneus and impaired performances of motor sequence learning [62]. In ALS, it has been suggested that cerebral hypermetabolism reflects neuroinflammation, characterized by activated astrocytes and microglia [16], rather than compensatory neuronal activity. Neuroinflammation has a deleterious effect and is therefore more consistent with the negative correlations we found in our patients between metabolic data and relevant cognitive scores.

This finding further highlights the potential role of cerebral hypermetabolism as a functional imaging marker for ALS.

### Voxelwise comparisons between alterations

This analysis revealed differences in the relative degrees of GM atrophy and cerebral glucose hypometabolism.

#### Greater atrophy than hypometabolism

Atrophy was greater than hypometabolism in two main regions: the temporal lobe (anterior, lateral and medial) and the calcarine sulcus. Based on previous studies carried out in Alzheimer’s disease, frontotemporal dementia or chronic alcoholism [18, 20, 63, 64], it is generally thought that loss of brain cells other than neurons might result in atrophy without associated hypometabolism. In ALS, an increasing number of studies have suggested that astrocytosis and/or microglial activation could be involved in the pathophysiology of the disease [65, 66].

The finding of a normal, i.e. higher than expected, level of glucose metabolism in atrophic cerebral zones may also suggest that compensatory mechanisms are at work in these structures, helping to maintain a moderately high metabolic level relative to structural alterations—a hypothesis previously developed for Alzheimer’s disease [18]. In this disease, according to the authors, the presence of abnormally phosphorylated tau proteins that aggregates to form neurofibrillary tangles may be one of the processes underlying severe atrophy and moderate hypometabolism in the hippocampus. In ALS, TDP-43 inclusions are located in neurons and astrocytes of ALS patients, not only in the motor regions but also in the temporal lobe [67]. Following the same reasoning, the presence of TDP-43 could lead to a massive neuronal loss, whereas moderate hypometabolism could be explained by a compensation of the remaining neurons.

#### Greater hypometabolism than GM atrophy

We found that hypometabolism was more severe than atrophy in the left superior medial frontal cortex. This result suggests that ALS is characterized by genuine functional alterations (metabolic, chemical or molecular) on top of the neuronal loss, heightening the functional consequences of local GM atrophy. Supporting the notion of metabolic alterations in ALS, a study used the benzodiazepine GABA_A_ marker ^11^C-flumazenil to study brain dysfunction in 17 patients [58]. Flumazenil is an antagonist at the benzodiazepine subunit of the GABA_A_ receptor. These authors found reduced binding of ^11^C-flumazenil in the dorsomedial prefrontal cortex notably. This may reflect the downregulation of postsynaptic GABA_A_ receptor expression [58]. Given that glucose metabolism reflects synaptic activity [68], the detrimental effects of ALS on neurotransmission systems could explain why metabolic dysfunction precedes GM atrophy (e.g. within the frontal lobe). Greater hypometabolism than GM atrophy has also been observed in the behavioural variant of FTD [20], suggesting that this could be a remote effect of GM atrophy on metabolism, or a diaschisis. *Diaschisis* refers to a change in the metabolic activity of neurons that are anatomically or functionally connected to a damaged area. The medial prefrontal cortex is connected to limbic structures such as the medial temporal lobe and putamen [69]. The neuronal loss reflected by GM atrophy within the temporal lobe and putamen (see above) may remotely affect the metabolism of the medial prefrontal cortex, though possibly only for a limited period of time, as disconnected neurons eventually die, giving rise to different patterns of brain volume and metabolic impairment.

The study has some limitations. The major one is the existence of two groups of healthy subjects. Indeed, as our protocol did not include the neuroimaging examinations for healthy subjects, we used the scans of healthy subjects of another protocol of our laboratory. Then, the threshold that we used in this article is relatively liberal even if it has been employed in several MRI [45, 49, 70–75] or PET studies [14, 16] in ALS. However, with a more stringent threshold, we could have missed some interesting findings. Finally, this study should be replicated in a bigger group of ALS patients.

## Conclusions

Taken together, our results confirm the existence of structural and metabolic changes in the brains of patients with ALS without dementia. We found that regional cerebral hypermetabolism is associated to impaired cognitive performance, which suggests that it reflects a local deleterious neuronal and/or astrocytic process. Our findings also emphasize the complex relationships between GM atrophy and hypometabolism, and the regional heterogeneity in their hierarchy. Greater GM atrophy than hypometabolism might mean either that brain tissue loss does not involve metabolically active cells or that the metabolism of the remaining cells is higher than expected. Greater hypometabolism than GM atrophy could reflect either a disconnection mechanism or an early stage of metabolic neuronal failure preceding cell death. Longitudinal studies are warranted to take account of the potential lapse between the different pathological processes.

## Abbreviations

ALS: Amyotrophic lateral sclerosis
ALS-FRS-R: ALS functional rating scale revised
CSF: Cerebrospinal fluid
FDG: ^18^F-fluorodeoxyglucose
FTD: Frontotemporal dementia
GM: Grey matter
MRI: Magnetic resonance imaging
PET: Positron emission tomography
PVE: Partial volume effect
SPM: Statistical parametric mapping
VBM: Voxel-based morphometry
WM: White matter
